# Contemporary intraoperative visualization for GBM with use of exoscope, 5-ALA fluorescence-guided surgery and tractography

**DOI:** 10.3171/2021.10.FOCVID21174

**Published:** 2022-01-01

**Authors:** Alexander J. Schupper, Jorge A. Roa, Constantinos G. Hadjipanayis

**Affiliations:** Department of Neurosurgery, Icahn School of Medicine at Mount Sinai, New York, New York

**Keywords:** 5-ALA, fluorescence-guided surgery, exoscope, high-grade glioma, neuronavigation, Raman spectroscopy

## Abstract

Maximal safe resection is the primary goal of glioma surgery. By incorporating improved intraoperative visualization with the 3D exoscope combined with 5-ALA fluorescence, in addition to neuronavigation and diffusion tensor imaging (DTI) fiber tracking, the safety of resection of tumors in eloquent brain regions can be maximized. This video highlights some of the various intraoperative adjuncts used in brain tumor surgery for high-grade glioma.

In this case, the authors highlight the resection of a left posterior temporal lobe high-grade glioma in a 33-year-old patient, who initially presented with seizures, word-finding difficulty, and right-sided weakness. They demonstrate the multiple surgical adjuncts used both before and during surgical resection, and how multiple adjuncts can be effectively orchestrated to make surgery in eloquent brain areas safer for patients. Patient consent was obtained for publication.

The video can be found here: https://stream.cadmore.media/r10.3171/2021.10.FOCVID21174

## Transcript

### 0:20

This video article shows the key technical aspects of the combined 3D exoscope with DTI tractography and 5-ALA fluorescence for high-grade glioma resection.

### 0:33

A 33-year-old male patient presents with refractory seizures following a prior brain biopsy consistent with a high-grade glioma. His neurological exam was initially significant for mild word-finding difficulties, otherwise nonfocal with no motor or sensory deficits and intact cranial nerves.

### 0:51

MRI revealed a left-sided multifocal tumor primarily located in the dominant superior temporal gyrus with extension to the supramarginal gyrus of parietal lobe.

### 1:03

The patient underwent a stereotactic biopsy, pathology consistent with an IDH1 wildtype glioblastoma (WHO grade IV). On POD 10, he was taken for a left frontotemporoparietal craniotomy for tumor resection. Given the proximity of the tumor to eloquent speech pathways, in addition to his clinical improvement allowing him to have fluent speech in both Russian and English, the surgical team elected to perform an awake craniotomy during the tumor resection portion of the case.

### 1:35

Preoperative planning was utilized with the Synaptive Medical software identifying the surgical corridor for the approach.

### 1:42

Preoperative MRI with diffusion tensor imaging (DTI) showing the relationship of the tumor to eloquent white matter tracts.

### 1:50

In this fly-through video, we can see the spatial distribution of the descending corticospinal tracts (blue), inferior longitudinal fasciculus (neon green), optic radiations (forest green), arcuate fasciculus (yellow), corpus callosum (red), cingulum (orange), uncinate fasciculus (pink), corona radiata (purple). Additionally, the tractography allows the surgical team to select the safest route of entry to the tumor.

### 2:23

Therefore, our surgical plan included (1) use of DTI tractography for understanding pathways surrounding the multifocal glioblastoma tumor; (2) performing an awake craniotomy and language mapping for safer resection due to the location of the tumor; (3) use of a voice-controlled 3D surgical exoscope for tumor debulking; and finally (4) administration of oral 5-ALA and use of fluorescence-guided surgery (FGS) for maximal resection at the tumor margin utilizing the new headlamp LED system for fluorescence visualization.

### 2:58

The patient was positioned supine with his head turned to the right in the Mayfield head clamp, to maximize access to the left temporal and parietal lobes. Synaptive navigation was used to plan our incision and tumor margins on the skin.

### 3:12

A trapdoor incision was made for the appropriate craniotomy for optimal visualization of the tumor tissue. Raney clips were used for superficial hemostasis, and fishhooks were attached to a Leyla bar to retract the skin flap.

### 3:26

The skull was removed using four burr holes parallel to the skin incision. 4-0 Nurolon tack-ups were used to close the epidural potential space.

### 3:37

The dura was excised in a C-shaped fashion and reflected inferiorly. The frontal and temporal lobes as well as the Sylvian fissure were identified.

### 3:48

The Synaptive 3D exoscope was brought into the field, and here we see the setup with the dual monitors, the left showing the 3D exoscopic view of the field, with the navigation probe in the resection cavity. On the right, you can see the navigation with the probe tip in the resection cavity. Additionally, the surrounding white matter tracts of interest that were designated prior to the case are highlighted.

### 4:12

Here you can see the highly vascular, high-grade glioma tumor tissue using the exoscope. There are several advantages to the use of the exoscope in tumor surgery. First, the operator is able to perform the surgery at a low light intensity (30% in this clip), causing less risk of tissue damage to the surrounding brain. There is a wide range of zoom variability allowing improved visualization of deep tissue structures, and the 3D wide-angle view allows greater depth perception not only for the operator but for observers as well, fostering a positive teaching environment for residents, fellows, and medical students.

### 4:51

The tumor tissue is removed and sent to pathology. Twenty tissue samples in total are taken as part of our clinical trial.

### 5:00

After tumor tissue is removed and the tumor core has been debulked, 5-ALA fluorescence-guided surgery is utilized.

### 5:06

5-ALA, a precursor metabolized in the heme biosynthesis pathway to protoporphyrin (PPIX), accumulates intracellularly in tumor cells and has a high affinity for high-grade glioma tissue. It is administered orally 4–6 hours prior to surgical resection and has a favorable safety profile. PPIX absorbs light between 375–440 nm and emits a violet-red fluorescence at 635 nm, which you can see here in this video. It is highly sensitive and specific for malignant brain tissue and can help us around the tumor margin to identify tumor from normal brain tissue. 5-ALA was used for the remainder of the tumor resection in this case.

### 5:52

Of note, we used a new LED headlamp system to visualize tumor fluorescence with surgical loupes; therefore, we did not need the microscope for fluorescence visualization.

### 6:07

Following tumor resection, meticulous hemostasis was achieved, and the surgical cavity was lined with Surgicel sheets.

### 6:16

The dura was closed in a watertight fashion.

### 6:19

And the bone flap was reapproximated with four burr hole covers.

### 6:23

The temporalis muscle was reapproximated, and the galea and skin were subsequently closed with suture.

### 6:29

Postoperative MRI shows a gross-total resection. The patient initially had some expressive aphasia following surgery, which improved over several days following surgery.

### 6:39

In summary, maximal safe resection is the primary goal of glioma surgery,^1^ and by incorporating improved intraoperative visualization with the 3D exoscope, 5-ALA fluorescence, neuronavigation, and diffusion tensor imaging (DTI) fiber tracking, we may maximize the safety of resection of tumors in eloquent brain regions.

### 6:58

Thank you.

## Disclosures

Dr. Hadjipanayis is a consultant for NXDC and Synaptive Medical Inc. He receives royalties from NXDC. He has also received speaker fees from Carl Zeiss and Leica.

## Author Contributions

Primary surgeon: Hadjipanayis. Assistant surgeon: Schupper. Editing and drafting the video and abstract: all authors. Critically revising the work: all authors. Reviewed submitted version of the work: all authors. Approved the final version of the work on behalf of all authors: Hadjipanayis. Supervision: Hadjipanayis.
